# Modeling Sensory Preference in Speech Motor Planning: A Bayesian Modeling Framework

**DOI:** 10.3389/fpsyg.2019.02339

**Published:** 2019-10-25

**Authors:** Jean-François Patri, Julien Diard, Pascal Perrier

**Affiliations:** ^1^Université Grenoble Alpes, CNRS, Grenoble INP, GIPSA-lab, Grenoble, France; ^2^Université Grenoble Alpes, CNRS, LPNC, Grenoble, France; ^3^Cognition Motion and Neuroscience Unit, Fondazione Istituto Italiano di Tecnologia, Genova, Italy

**Keywords:** speech motor control, Bayesian modeling, sensory integration, sensory preference, speech motor goals

## Abstract

Experimental studies of speech production involving compensations for auditory and somatosensory perturbations and adaptation after training suggest that both types of sensory information are considered to plan and monitor speech production. Interestingly, individual sensory preferences have been observed in this context: subjects who compensate less for somatosensory perturbations compensate more for auditory perturbations, and *vice versa*. We propose to integrate this sensory preference phenomenon in a model of speech motor planning using a probabilistic model in which speech units are characterized both in auditory and somatosensory terms. Sensory preference is implemented in the model according to two approaches. In the first approach, which is often used in motor control models accounting for sensory integration, sensory preference is attributed to the relative precision (i.e., inverse of the variance) of the sensory characterization of the speech motor goals associated with phonological units (which are phonemes in the context of this paper). In the second, “more original” variant, sensory preference is implemented by modulating the sensitivity of the comparison between the predicted sensory consequences of motor commands and the sensory characterizations of the phonemes. We present simulation results using these two variants, in the context of the adaptation to an auditory perturbation, implemented in a 2-dimensional biomechanical model of the tongue. Simulation results show that both variants lead to qualitatively similar results. Distinguishing them experimentally would require precise analyses of partial compensation patterns. However, the second proposed variant implements sensory preference without changing the sensory characterizations of the phonemes. This dissociates sensory preference and sensory characterizations of the phonemes, and makes the account of sensory preference more flexible. Indeed, in the second variant the sensory characterizations of the phonemes can remain stable, when sensory preference varies as a response to cognitive or attentional control. This opens new perspectives for capturing speech production variability associated with aging, disorders and speaking conditions.

## 1. Introduction

The recent history of research that investigates the links between phonology, production and perception of speech has been marked by vigorous exchanges between proponents of purely acoustic/auditory theories (Stevens, 1972; Stevens and Blumstein, 1978; Blumstein and Stevens, 1979; Lindblom, 1990; Sussman et al., 1991) for whom the physical correlates of phonological units would be exclusively in the acoustic domain, and proponents of theories who rather saw these correlates primarily in the articulatory/somatosensory domain (Fowler, 1986; Saltzman, 1986). These debates were all the more vigorous because they were related to important theoretical issues around phonological theories (Chomsky and Halle, 1968; Clements, 1985; Keyser and Stevens, 1994 vs. Browman and Goldstein, 1989, 1992; Goldstein and Fowler, 2003) and cognitive theories of perception (Diehl and Kluender, 1989 vs. Gibson, 1979 vs. Liberman et al., 1967).

As a consequence, models that were designed to simulate and investigate the process of articulation and sound production from the specification of phonological sequences (we will call these models Speech Production Models henceforth) were split into two main categories: models in which the goals of the speech task were specified in the articulatory domain (Coker, 1976; The Task Dynamics Model: Kelso et al., 1986; Saltzman and Munhall, 1989; The DIVA Model Version 1: Guenther, 1995; Kröger et al., 1995; The C/D model: Fujimura, 2000), and models in which the goals were specified in the acoustic domain (The DIVA Model Version 2: Guenther et al., 1998; GEPPETO: Perrier et al., 2005).

A number of experimental studies have been carried out in order to find clear support for one or the other of these theories. The majority of them relied on perturbation paradigms, in which one of the modalities, either acoustic or articulatory, was perturbed. Patterns of behavioral adaptation to perturbation of the jaw with bite-blocks (Gay et al., 1981) or of the lips with lip-tubes (Savariaux et al., 1995) were interpreted as evidence for the specification of the goal in the acoustic/auditory domain, whereas adaptation in response to a perturbation of the jaw with a velocity-dependent force field (Tremblay et al., 2003) supported the hypothesis of a goal in the articulatory/somatosensory domain. In the absence of any evidence supporting undeniably one of these theories, new theories emerged assuming that phonological units could be associated with both auditory and somatosensory goals (see for example the concept of “perceptuo-motor unit” in the Perception-for-Action-Control Theory of Schwartz et al. (2012); or, for another perspective, the phonological processing of the HFSC model of Hickok (2012) distributed over an auditory-motor circuit for syllable and over a somatosensory-motor circuit for the phonemes).

Today, the large majority of the Speech Production Models associate both somatosensory and auditory goals to phonological units (Guenther et al., 2006; Kröger et al., 2009; Hickok, 2012; Yan et al., 2014; Parrell et al., 2018). In this context, a key-question is the respective weight of each modality in the specification of the goals. Lindblom (1996) and Stevens (1996) considered that the articulatory/somatosensory correlates are not primary, but are rather the secondary consequences of the articulatory strategies that have emerged for a correct achievement of the acoustic/auditory goals. In line with these suggestions, we have assumed a hierarchical organization of the goals, with a higher priority for the achievement of the auditory goals (Perrier, 2005). In its recent versions, the DIVA model assumes that speech acquisition is based on purely auditory targets, and that the somatosensory targets are learned in a second stage during speech development as “sensations associated with the sound currently being produced” (Guenther et al., 2006, p. 286), introducing also a hierarchy in the role of the modalities in the specification of the goals. In an experimental study, in which speech production was perturbed both in the auditory domain (with an on-line shift of formant F1) and in the somatosensory one (with an on-line alteration of the jaw opening, which also affects F1), Feng et al. (2011) found that participants compensated for the auditory perturbation regardless of the direction of the perturbation of the jaw opening. This observation was in support of a dominant role of the auditory modality in the control of speech production.

However, three important experimental findings have contested the validity of the hierarchical hypothesis. The first finding is the fact that, when the auditory feedback is perturbed, the compensation to the perturbation is never complete, with a magnitude commonly being at the most at 1/3 of the perturbation (Houde and Jordan, 2002; Purcell and Munhall, 2006; Villacorta et al., 2007; Cai et al., 2010). A convincing explanation for this phenomenon is the fact that the strength of the specification of the somatosensory goal limits the authorized magnitude of the articulatory changes used to compensate for the auditory perturbation (Villacorta et al., 2007; Katseff et al., 2012). The second finding is that motor learning associated with a perturbation of the auditory feedback generates a shift of the perceptual boundaries between the phonemes of interest (Shiller et al., 2009; Lametti et al., 2014). Using a simplified Bayesian model of speech production, we have shown that the perceptual boundary shift was also in part due to the strength of the somatosensory goals (Patri et al., 2018). The third finding is the observation of “sensory preference” in a speech production task in which both auditory feedback and jaw movement were perturbed on line (Lametti et al., 2012). Indeed Lametti et al. (2012) found that contrary to the observations of Feng et al. (2011) not all the participants did compensate in priority for the auditory perturbation: some of them did compensate more for the auditory perturbation, but some others did compensate more for the jaw perturbation, and a significant negative correlation was found between the amounts of compensation to the perturbation in each modality. This completely changed the way to consider the crucial question of the physical domain in which the speech goals are specified in adults speakers for the production of phonological units. The answer to this question would not be generic and only depending on the characteristics of the language, but would be strongly subject-dependent and related to a preference of the subjects for one feedback modality or the other. From a general linguistic point of view, the debate currently moves toward considering speaker-specific characteristics of the way to deal with the constraints of the language. Developing models of such phenomena will open doors for the elaboration of new experimental paradigms to question how speakers deal with the constraints of their language, and to investigate the consequences on speaker behaviors in terms of adaptation, coarticulation, and possibly diachronic phonetic changes.

In this work, we address the question of the “sensory preference” within a Bayesian model of speech motor planning, in which speech units are characterized both in auditory and somatosensory terms. This approach includes internal models predicting the sensory consequences of motor commands, and the definition of the sensory characterization of the motor goals, also called henceforth “sensory targets,” associated with phonemes. These components are described in terms of probability distributions. We show that sensory preference can be implemented in the model in two ways.

In the first variant, sensory preference is attributed to the relative accuracy measured as the precision (i.e., inverse of variance) of the sensory targets. This is inspired from well-acknowledged models of sensory fusion for perception (Ernst and Banks, 2002; Alais and Burr, 2004; Kersten et al., 2004) and of sensorimotor integration (Körding and Wolpert, 2004). It corresponds in particular to the approach proposed by the DIVA model (Villacorta et al., 2007; Perkell et al., 2008). In this view, sensory preference originates from the level of the stored sensory targets that are intrinsically associated with phonological units. This suggests that sensory preference would be an inflexible property of each individual. We call this modeling approach “Target-based approach.”

In the second, more original variant, sensory preference is implemented by modulating the sensitivity of the comparison between the predicted sensory consequences of motor commands and the sensory characterization of speech motor goals. This approach differs from linear weightings of the error associated with each modality in the computation of the feedback correction signal (see for example the “synaptic weights” in Guenther et al., 2006, Equation 9, p. 286), because of our probabilistic formulation. Indeed, we will see that the probabilistic formulation enables an interesting interpretation of the variation of sensory preference in terms of “clarity” or “sharpness” of the sensory pathway. Furthermore, in this second view, sensory preference is more flexible, as it can be modified without changing the stored sensory targets. Such a modification can then result from cognitive control, attentional processes or features of the task, without affecting the sensory characterization of speech motor goals associated with phonological units. We call this modeling approach “Comparison-based approach.”

The main purpose of the current study is to compare these two variants, in the context of the adaptation to a long-lasting steady-state external sensory perturbation. As we recalled above, numerous experimental studies have used such a perturbation paradigm, and they have shown that perturbation leads to two kinds of compensation depending on the exposure time to the perturbation: first to an almost immediate change of speech articulation aiming at compensating for the unpredicted newly introduced perturbation; second, after a sufficiently long period in presence of the sustained perturbation, to a long-lasting compensation resulting from adaptation. Adaptation has been shown to induce after-effects (Houde and Jordan, 1998; Tremblay et al., 2003) which has been interpreted as evidence for long-lasting changes in the internal representations of the relations between motor commands and sensory outputs (called internal models in this paper). Thus, it is important to distinguish immediate compensation, associated with instantaneous motor control of speech movements, and compensation resulting from adaptation, associated with changes in the planning of speech movements. In this work we focus on the compensation resulting from adaptation, without considering the dynamics of the learning process underlying the transition from immediate compensation to final adaptation.

This paper is structured as follows. In section 2, we introduce all the elements of the modeling framework. We first describe the GEPPETO model, overall, and detail the Bayesian version of its motor planning layer. Then we explain how we simulate sensory perturbations and how we account for the resulting adaptations. Finally, we describe both variants of our model of sensory preference. In section 3, we simulate the two variants, highlighting their equivalence, which we then analyze formally. Finally, we discuss our results and possible extensions in section 4.

## 2. Methods

### 2.1. Overview of the Framework

#### 2.1.1. The GEPPETO Model

**Figure 1:**
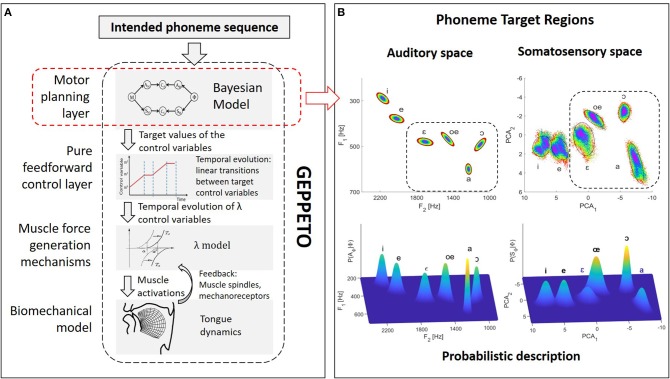
Schematic representation of the GEPPETO model. **(A)** Overview of the four layers of the GEPPETO model. The red dashed box indicates the planning layer on which we focus in the present work and which is the object of the Bayesian modeling. **(B)** Illustration of phoneme sensory target regions in the model. Top plots: ellipses representing auditory target regions in the (*F*_2_, *F*_1_) acoustic plane (left) and in the first two PCA dimensions of the somatosensory space (right). Colors enable to visualize the distortion of geometry induced by the non-linearity of the relation between the auditory and somatosensory spaces. Dashed boxes indicate the portion of auditory and somatosensory spaces on which we focus for the results presented in this paper. Bottom plots: probabilistic characterization of phoneme target regions in the Bayesian model as multivariate Gaussian distributions.

GEPPETO (see Figure 1) is a model of speech production organized around four main components: (i) a biomechanical model of the vocal tract simulating the activation of muscles and their influence on the postures and the movements of the main oro-facial articulators involved in the production of speech (Perrier et al., 2011); (ii) a model of muscle force generation mechanisms (the λ model, Feldman, 1986) that includes the combined effects on motoneurons' depolarization of descending information from the Central Nervous System and afferent information arising via short delay feedback loops from muscle spindles (stretch reflex) or mechano-receptors; (iii) a pure feedforward control system that specifies the temporal variation of the control variables (called λ variables) of the λ model from the specification of the target values inferred in the motor planning phase and of their timing; and (iv) a motor planning system that infers the target λ variables associated with the phonemes of the planned speech sequence.

In the implementation of GEPPETO used in this study, the biomechanical model is a 2-dimensional finite element model of the tongue in the vocal tract, which includes 6 principal tongue muscles as actuators and accounts for mechanical contacts with the vocal tract boundaries. The motor planning layer specifies the target λ variables by considering the motor goals associated with the phonemes of the speech utterance to be produced and using an optimal approach. Complete descriptions of GEPPETO, available elsewhere (Perrier et al., 2005; Winkler et al., 2011; Patri et al., 2015, 2016; Patri, 2018), also involve the specification of intended levels of effort. This enables in particular to perform speech sequences at different speaking rates; however, for simplicity, we do not consider this aspect of the model in the current study.

A key hypothesis in GEPPETO is that speech production is planned on the basis of units having the size of the phonemes. The account for larger speech units is given in the model via optimal planning: larger speech units correspond to the span of the phoneme sequence on which optimal planning applies (CV syllables, CVC syllables, VCV sequences, see Perrier and Ma, 2008; Ma et al., 2015). Given the limitations of the biomechanical model used in this study, which only models the tongue and assumes fixed positions for the jaw and the lips, we only consider French vowels that do not crucially involve jaw or lip movements, which are {/i/, /e/, /ɛ/, /a/, /oe/, /ɔ/}. GEPPETO further assumes that the motor goals associated with phonemes are defined as particular target regions in the sensory space. These regions are assumed to describe the usual range of variation of the sensory inputs associated with the production of the phonemes. Previous versions of GEPPETO have only considered the auditory space for the definition of these target regions. The auditory space is identified in GEPPETO to the space of the first three formants (F_1_, F_2_, F_3_) and target regions are defined in this space as dispersion ellipsoids of order 2, whose standard-deviations have been determined from measures provided by phoneme production experiments (Calliope, 1984; Robert-Ribes, 1995; Ménard, 2002) and adapted to the acoustic maximal vowel space of the biomechanical model (Perrier et al., 2005; Winkler et al., 2011). The top left part of Figure 1B represents the projection of these target regions in the (*F*_2_, *F*_1_) plane.

In the present study, we consider an updated version of GEPPETO that includes both auditory and somatosensory characterizations of the phonemes. We call it “Bayesian GEPPETO,” because the planning layer, which is at the core of the present study, is described with a Bayesian model. In this formulation, the somatosensory space only accounts for tongue proprioception. This account is based on the shape of the tongue contour in the mid-sagittal plane. More specifically, the somatosensory space is defined as the space of the first three Principal Components that model the covariation of the 17 nodes of the tongue contour in the Finite Element tongue mesh in the mid-sagittal plane, when the target λ variables vary over a large range of values, which covers all possible realistic tongue shapes associated with vowel productions. In line with the idea that auditory goals are primary in speech acquisition and that somatosensory goals are learned as a consequence of the achievement of the auditory goals (Lindblom, 1996; Stevens, 1996; Guenther et al., 2006), GEPPETO assumes that somatosensory target regions characterizing phonemes are dispersion ellipsoids that approximate the projections of the auditory target regions into the somatosensory space. The top right part in Figure 1B illustrates the somatosensory target regions in the plane of the first two principal components. Data points within increasing elliptical rings in the auditory target regions are plotted with identical colors in the auditory and somatosensory spaces, providing an intuitive idea of the geometry distortion resulting from the non-linear relation between the auditory and the somatosensory space.

For a given phoneme sequence, the goal of the motor planning layer of GEPPETO is to find the λ target variables that enable to reach the sensory target regions of the phonemes with the appropriate serial-order. In the most recent developments of GEPPETO, this inverse problem is addressed as an inference question formulated in a Bayesian modeling framework (Patri et al., 2015, 2016). It is on this Bayesian component of GEPPETO that we focus in this work.

#### 2.1.2. Bayesian Modeling of Speech Motor Planning in GEPPETO

The Bayesian model formulates the key ingredients of the motor planning stage of GEPPETO in a probabilistic framework, where key quantities are represented as probabilistic variables and their relations are represented by probability distributions. It is mathematically based on the theoretical concepts defined in the COSMO model of speech communication (Moulin-Frier et al., 2015; Laurent et al., 2017). In previous works we have described our modeling framework in the context of coarticulation modeling, planning of sequences of phonemes (Patri et al., 2015), and the specification of effort levels for the planning of speech at different speaking rates (Patri et al., 2016). However, these previous implementations of the model only considered auditory goals for the phonemes. A novelty in the present work is the integration of both auditory and somatosensory goals in “Bayesian GEPPETO.” This integration is based on modeling principles that we have recently elaborated in the context of a simplified Bayesian model of speech production (Patri et al., 2018), in the aim to study various potential explanations for the shifts of perceptual boundaries observed after speech motor learning (Shiller et al., 2009; Lametti et al., 2014). Note that for simplicity we focus here only on the production of single phonemes. However, the extension of the present formulation to consider sequences of phonemes as in Patri et al. (2015) is straightforward.

**Figure 2:**
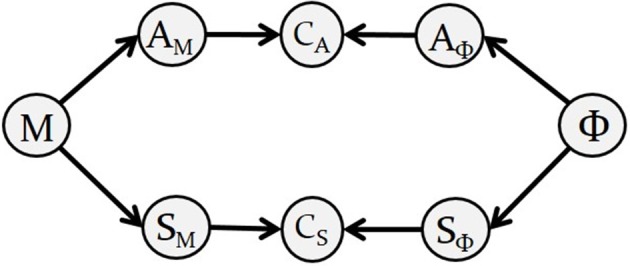
Diagram describing the Bayesian representation of the motor planning layer in GEPPETO. Nodes represent variables in the model and arrows represent their dependencies. The diagram is a graphical representation of the decomposition of the joint probability distribution given in Equation (1).

In the case of single-phoneme planning, “Bayesian GEPPETO” includes eight probabilistic variables, described in Figure 2 along with their dependencies. The right hand side of the diagram represents variables involved in the definition of the motor goals associated with phonemes: variable Φ is the variable representing phoneme identity, variables *A*_Φ_ and *S*_Φ_ are auditory and somatosensory variables involved in the sensory characterization of phonemes (we call them sensory-phonological variables). The left hand side of the diagram represents variables involved in sensory-motor predictions: the 6-dimensional motor control variable *M* represents the six λ variables that control muscle activation and then tongue movements in the biomechanical model (*M* = (λ_1_, …, λ_6_)); variables *A*_*M*_ and *S*_*M*_ are sensory-motor variables representing the auditory and somatosensory consequences of motor variable *M*.

Motor planning of a single phoneme is achieved in the model by identifying the sensory-motor predictions that match the sensory specification of the intended phoneme. This matching is imposed with two coherence variables *C*_*A*_ and *C*_*S*_ (Bessière et al., 2013), that act as “probabilistic switches,” and can be understood as implementing a matching constraint between the predicted sensory-motor variables and the specified sensory-phonological variables.

The diagram in Figure 2 also represents the decomposition of the joint probability distribution of all the variables in the model:

(1) $$\begin{array}{l} \;P\left(M\;\Phi A_{M}A_{\Phi }C_{A}S_{M}S_{\Phi }C_{S}\right)=P\left(M\right)P\left(\Phi \right)\\ \begin{array}{c} P\left(A_{M}\;|M\right)P\left(A_{\Phi }\;|\;\Phi \right)P\left(C_{A}\;|A_{M}A_{\Phi }\right)\\ \begin{array}{c} P\left(S_{M}\;|M\right)P\left(S_{\Phi }\;|\;\Phi \right)P\left(C_{S}\;|S_{M}S_{\Phi }\right)\;. \end{array} \end{array} \end{array}$$

Each of the factors on the right hand side of Equation (1) corresponds to one particular piece of knowledge involved in motor planning:

*P*(*M*) and *P*(Φ) are prior distributions representing prior knowledge about possible values of motor variable *M* and of phoneme variable Φ. We assume all possible values to be equally probable (no prior knowledge) and thus define *P*(*M*) and *P*(Φ) as uniform distributions over their domains. The domain of variable *M* is a continuous 6-dimensional support defined by the allowed range of values of each parameter λ_*i*_ of the biomechanical model. Φ is a discrete, categorical variable including the identity of the different phonemes considered in the model.
*P*(*A*_Φ_ | Φ) and *P*(*S*_Φ_ | Φ) correspond to the auditory and somatosensory characterizations of phonemes. We define them as multivariate Gaussian distributions in the auditory and somatosensory spaces:
  (2) $$\begin{array}{r} P\left(\left[X_{\Phi }=x\right]\;|\;\left[\Phi =\phi \right]\right):=N\left(x\;;\;\mu_{X}^{\phi },\Gamma_{X}^{\phi }\right), \end{array}$$
where *X* refers to the sensory modality (*A* for “Auditory” or *S* for “Somatosensory”), and $\mu_{X}^{\phi }$ and $\Gamma_{X}^{\phi }$ correspond to the parameters specifying the distribution associated to phoneme ϕ in the sensory space *X* (i.e., mean vector $\mu_{X}^{\phi }$ and covariance matrix $\Gamma_{X}^{\phi }$). This definition of the sensory characterizations translates in probabilistic terms the hypothesis that phonemes are characterized by the ellipsoid regions illustrated in Figure 1B. In particular, the mean vector and covariance matrix of each distribution are identified from these ellipsoid regions. The correspondence between these two representations is illustrated in the top and bottom plots of Figure 1B.
*P*(*A*_*M*_ | *M*) and *P*(*S*_*M*_ | *M*) correspond to the knowledge relating the motor control variable *M* to its predicted sensory consequences *A*_*M*_ and *S*_*M*_, in the auditory and somatosensory space, respectively. We identify this knowledge to sensory-motor internal models in the brain (Kawato et al., 1990; Jordan and Rumelhart, 1992; Tian and Poeppel, 2010). In the current implementation we assume that these internal models are deterministic and we implement them as Dirac probability distributions centered on the outputs of sensory-motor maps, ρ_*a*_ and ρ_*s*_:
  (3) $$\begin{array}{r} P\left(\left[X_{m}=x\right]\;|\;\left[M=m\right]\right):=\delta \left(x-\rho_{x}\left(m\right)\right)\;, \end{array}$$
where *X*_*m*_ stands for *A*_*M*_ or *S*_*M*_, depending on the modality, δ denotes the Dirac distribution (i.e., *P*([*X*_*M*_ = *x*] | [*M* = *m*]) is zero unless *x* = ρ_*x*_(*m*)). The sensory-motor maps ρ_*a*_ and ρ_*s*_ have been created from the results of around 50,000 simulations carried out with the biomechanical model by randomly sampling the space of the λ motor control variables. We implemented these sensory maps by learning the relation between the λ variables and the sensory variables with Radial Basis Functions (RBF; Poggio and Girosi, 1989) with a usual supervised learning approach.
*P*(*C*_*A*_ | *A*_*M*_*A*_Φ_) and *P*(*C*_*S*_ | *S*_*M*_*S*_Φ_) implement the two sensory matching constraints. *C*_*A*_ and *C*_*S*_ are both binary variables (taking values 0 or 1) that activate the corresponding matching constraint when their values are set to 1. This is implemented with the following definition:
  (4) $$P([C_{X}=1]|[X_{M}=x_{m}]\;[X_{\Phi }=x_{\phi }])\;:=\{\begin{array}{ll} 1 & \text{if }x_{m}=x_{\phi }\\ 0 & \text{otherwise}. \end{array}$$
where again *X*_*M*_ stands for *A*_*M*_ or *S*_*M*_, and *X*_Φ_ stands for *A*_Φ_ or *S*_Φ_.

#### 2.1.3. Motor Planning in the Bayesian Model

The goal of the motor planning layer in GEPPETO is to find values of the motor control variable *M* that correctly make the tongue articulate the intended phoneme. The Bayesian model enables to address this question as an inference question that can be formulated in three ways: (i) by activating only the auditory pathway with [*C*_*A*_ = 1]; (ii) by activating only the somatosensory pathway with [*C*_*S*_ = 1]; (iii) by activating both the auditory and somatosensory pathways with [*C*_*A*_ = 1] and [*C*_*S*_ = 1] (we call this the “fusion” planning model). These three planning processes are computed analytically, by applying probabilistic calculus to the joint probability distribution *P*(*MA*_*M*_*S*_*M*_*A*_Φ_*S*_Φ_ Φ*C*_*A*_*C*_*S*_) specified by Equation (1). The outcome of these computations for each planning process gives:

(5) $$\begin{array}{r} P\left(\left[M=m\right]\;|\Phi \left[C_{A}=1\right]\right)\propto P\left(\left[A_{\Phi }=\rho_{a}\left(m\right)\right]\;|\;\Phi \right), \end{array}$$

(6) $$\begin{array}{r} P\left(\left[M=m\right]\;|\Phi \left[C_{S}=1\right]\right)\propto P\left(\left[S_{\Phi }=\rho_{s}\left(m\right)\right]\;|\;\Phi \right), \end{array}$$

(7) $$\begin{array}{l} P\left(\left[M=m\right]\;|\Phi \left[C_{A}=1\right]\;\left[C_{S}=1\right]\right)\propto P\left(\left[A_{\Phi }=\rho_{a}\left(m\right)\right]\;|\Phi \right)\\ \begin{array}{c} P\left(\left[S_{\Phi }=\rho_{s}\left(m\right)\right]\;|\Phi \right), \end{array} \end{array}$$

where the mathematical symbol “∝” means “proportional to.”

**Figure 3:**
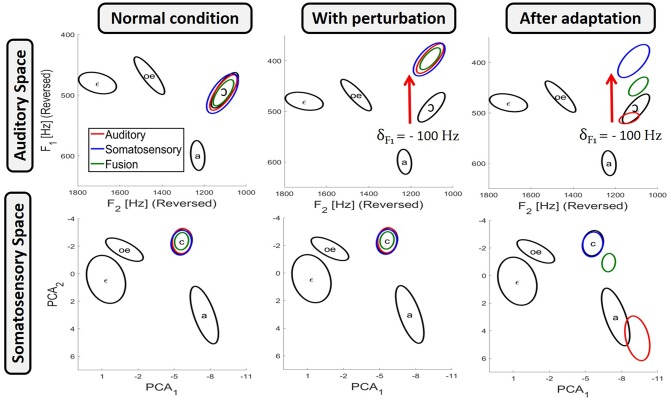
Results of the three planning processes obtained with the model for the production of phoneme /ɔ/, in the auditory space (top panels) and the somatosensory space (bottom panels). Results are presented in three conditions: in unperturbed condition (left panels); with auditory perturbation before adaptation (middle panels); and with auditory perturbation once adaptation has been achieved (right panels). Black ellipses indicate the phoneme target regions (see Figure 1B). Colored ellipses present results as dispersion ellipses of order 2 obtained from 2.10^4^ samples for each of the three planning processes: auditory planning in red, somatosensory planning in blue and fusion planning in green.

Equations (5–7) give the probability, according to each of the three planning process, that a given value *m* of the motor control variable *M* will actually produce the intended phoneme Φ. Practically, in order to have for each planning process a reasonable set of values covering the range of variation of the motor control variable with their probability to correctly produce the intended phoneme, we randomly sampled the space of the motor control variable according to these probability distribution. This sampling was implemented to approximate the probability distributions with a standard Markov Chain Monte Carlo algorithm (MCMC) using Matlab's “mhsample” function. The MCMC algorithm performs a random walk in the control space resulting in a distribution of random samples that converges toward the desired probability distribution. The left panels in Figure 3 present the dispersion ellipses of order 2 in the auditory and somatosensory spaces of the result obtained from 2.10^4^ random samples, taken from 20 independent sampling runs (after removal of the first 10^3^ burn-in samples in each chain), for the production of phoneme /ɔ/ for each of the three planning processes. It can be observed that all three planning processes correctly achieve the target region in both sensory spaces.

### 2.2. Implementation of Sensory Perturbations and Adaptation in the Model

Sensory perturbations alter the sensed consequence of motor actions such that the sensory output predicted by the internal model becomes erroneous. When the perturbation is consistently maintained, a new relation between motor control variables and sensory outputs is experienced and the sensory-motor internal models (*P*(*A*_*M*_ | *M*) and *P*(*S*_*M*_ | *M*)) are updated as a result of motor learning and adaption (Shadmehr and Mussa-Ivaldi, 1994; Houde and Jordan, 1998; Haruno et al., 1999; Tremblay et al., 2003), in order to capture the new sensory-motor relation imposed by the perturbation. We define adaptation, in the model, as the update of the parameters of the internal models.

According to Lametti et al. (2012), differences in sensory preference lead to differences across speakers in their tolerance to errors in each of the sensory modalities (auditory or somatosensory). This phenomenon has been assumed to explain the observed inter-speaker differences in the amount of compensation after adaptation. The evaluation of our two implementations of sensory preference is based on their capacity to account for these differences in compensation. Importantly, whatever the nature of the sensory perturbation (auditory or somatosensory), compensation induces changes in both the auditory and somatosensory outputs, generating errors in both domains. Hence, the amount of compensation is modulated by sensory preference even if the perturbation affects only one sensory modality. Therefore in this paper, for the sake of simplicity, we only consider auditory perturbations (but see Patri, 2018 for results involving somatosensory perturbations).

#### 2.2.1. Implementation of Sensory Perturbations

We simulate auditory perturbations in the model by altering the spectral characteristic of the acoustic signal associated with the tongue configurations of the biomechanical model. More specifically, if a tongue configuration *T* produced an acoustic output *a*^*u*^ in unperturbed condition, then with the auditory perturbation the same tongue configuration will result in a shifted acoustic output a^*^ = *a*^*u*^+δ. The middle panel of Figure 3 illustrates the effect of an auditory perturbation that shifts the first formant F1 down by δ = −100 Hz, during the production of vowel /ɔ/ for the three planning processes.

#### 2.2.2. Implementation of Adaptation

In the context of an auditory perturbation, only the auditory-motor internal model *P*(*A*_*M*_ | *M*) becomes erroneous. Hence, we implement adaptation to the auditory perturbation by updating the auditory-motor map ρ_*a*_ of the auditory-motor internal model *P*(*A*_*M*_ | *M*) (see Equation 3). This update is defined in order to capture the new relation between the motor control variable and its auditory consequence. In the case of an auditory perturbation that shifts auditory values by a constant vector δ, we assume the resulting update to be complete and perfect, of parameter δ_*A*_ = δ:

(8) $$\begin{array}{r} \rho_{a}^{*}\left(m\right)=\rho_{a}^{u}\left(m\right)+\delta_{A}. \end{array}$$

where $\rho_{a}^{*}$ and $\rho_{a}^{u}$ denote the auditory-motor maps in the perturbed and unperturbed condition, respectively. In all simulations involving the perturbation, we choose to shift only the first formant F1 down by −100 Hz, such that δ_*A*_ = [−100, 0, 0].

The right panel of Figure 3 illustrates the effect of the auditory perturbation and the outcome of adaptation for each of the three planning processes. In unperturbed conditions (left panels), all three planning processes correctly achieve both the auditory and the somatosensory target regions. In the middle panel, which represents the situation before adaptation occurs, the auditory perturbation induces for the three planning processes a shift in the auditory domain (top middle panel), and obviously not in the somatosensory domain (bottom middle panel), since the perturbation only alters the auditory-motor relations. The right panels illustrate the outcome of the three planning processes after adaptation has been achieved, as implemented by Equation (8). It can be seen that the results corresponding to the somatosensory planning, *P*(*M* | Φ [*C*_*S*_ = 1]), remain unchanged. This is because somatosensory planning does not involve the auditory-motor map ρ_*a*_ (Equation 6), and is then not concerned by the update of the auditory-motor map induced by the adaptation. On the other hand, and as expected, after the perfect update of the auditory-motor internal model, the auditory planning *P*(*M* | Φ [*C*_*A*_ = 1]) (Equation 5) fully compensates for the perturbation and results in a correct reaching of the auditory target region (top right panel). However, this compensation is achieved by a change in the value of the motor control variable, which results in a tongue posture associated with a somatosensory output that is outside of the somatosensory target region (bottom right panel). Finally, the fusion planning *P*(*M* | Φ [*C*_*A*_ = 1] [*C*_*S*_ = 1]) (Equation 7) combines the two previous results: since auditory and somatosensory target regions are no more compatible due to the update of the auditory-motor internal model, fusion planning cannot reach both sensory target regions at the same time, and therefore it makes a compromise between the auditory and the somatosensory constraints. As a result, fusion planning leads to auditory and somatosensory consequences that lie midway between those of a pure auditory or a pure somatosensory planning.

In summary, we have described how the three planning processes achieve similar results in unperturbed condition but generate very different results after adaptation to the sensory perturbation. Intuitively, if we are able to modulate in the model the weight associated with each sensory modality in the fusion planning process, we would be able to achieve a continuum of compensation magnitudes after adaptation. This continuum, representing all the possible patterns of sensory preference, would go from full compensation for the auditory perturbation, when sensory preference induces a full reliance on the auditory modality, to no compensation at all when sensory preference induces a full reliance on the somatosensory modality.

For the evaluation of the two variants of our model of sensory preference, we mainly consider the “fusion” planning, as it is the planning process that combines both auditory and somatosensory pathways, and then enables an account of the sensory preference phenomenon (see Equation 7). However, we will also study the planning processes based on each sensory pathway individually, in order to have them as reference to evaluate the consequences of different sensory preference patterns. The impact of sensory preference on planning will be evaluated by modulating the relative involvement of each sensory pathway in the planning process. In general terms, the involvement of a sensory pathway is related to the magnitude of the mismatch between sensory-motor predictions and the intended target: for example, by increasing the magnitude of this mismatch for the auditory modality we obtain an increase of the involvement of auditory pathway in the planning process.

### 2.3. Modeling Sensory Preference

#### 2.3.1. The Target-Based Approach: Modulating the Precision of Sensory Targets

**Figure 4:**
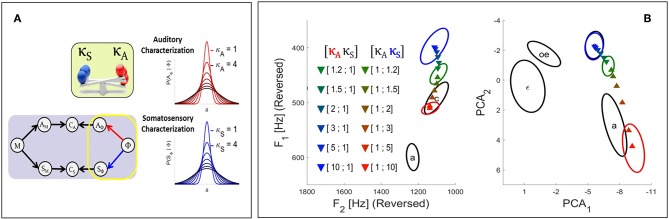
**(A)** Illustration of the effect in the Target-based approach of parameters κ_*A*_ and κ_*S*_ (see text) on the auditory and somatosensory target regions associated with phonemes *P*(*A*_Φ_ | Φ) and *P*(*S*_Φ_ | Φ). The greater the value of κ parameter, the wider the target region, and the weaker the contribution of the corresponding sensory pathway to the planning process. **(B)** Results of the fusion planning process after adaptation to the auditory perturbation described in section 2.2.2, for different values of parameters κ_*A*_ and κ_*S*_.

In the Target-based approach we modulate the involvement of each sensory modality at the level of the target regions associated with phonemes, as illustrated in the left panel of Figure 4. In our model, the target regions result from the sensory characterization of phonemes which is represented by the terms *P*(*A*_Φ_ | Φ) and *P*(*S*_Φ_ | Φ). These terms are specified in Equation (2) as multivariate Gaussian probability distributions with mean vectors $\mu_{A}^{\Phi }$ and $\mu_{S}^{\Phi }$ and covariance matrices $\Gamma_{A}^{\Phi }$ and $\Gamma_{S}^{\Phi }$, respectively. We implement sensory preference in the model by modulating the precision of these distributions with the introduction of two additional parameters, respectively κ_*A*_ and κ_*S*_ for the auditory and the somatosensory pathway. These parameters multiply the covariance matrices of the corresponding Gaussian distributions:

(9) $$\begin{array}{r} P\left(\left[X_{\Phi }=x\right]|\left[\Phi =\phi \right]\right)=N\left(x\;;\;\mu_{X}^{\phi },\;\kappa_{X}\Gamma_{X}^{\phi }\right), \end{array}$$

where *X*, once more, stands either for the auditory or the somatosensory modality. The left panel of Figure 4 illustrates the effect of parameters κ_*X*_ on the target distributions in a one-dimensional case: increasing κ_*X*_ results in widening the distribution, and as suggested previously this induces a decrease of the involvement of the corresponding sensory modality in the planning process, since larger distributions will less penalize sensory signals that depart from the center of the target region and will thus allow larger errors in this sensory modality. The same reasoning applies to a decrease of κ_*X*_, which will induce a narrowing of the distribution and an increase of the involvement of the corresponding sensory modality.

Replacing the forms given by Equation (9) into Equation (7) gives a first formulation of the influence of sensory preference in the fusion planning process:

(10) $$\begin{array}{l} P\left(\left[M=m\right]\;|\Phi \left[C_{A}=1\right]\left[C_{S}=1\right]\right)\\ \begin{array}{c} \propto N\left(\rho_{s}\left(m\right)\;;\;\mu_{S}^{\Phi },\;\kappa_{S}\Gamma_{S}^{\Phi }\right)N\left(\rho_{a}\left(m\right)\;;\;\mu_{A}^{\Phi },\;\kappa_{A}\Gamma_{A}^{\Phi }\right), \end{array} \end{array}$$

#### 2.3.2. The Comparison-Based Approach: Modulating the Weight of the Sensory Matching Constraints

**Figure 5:**
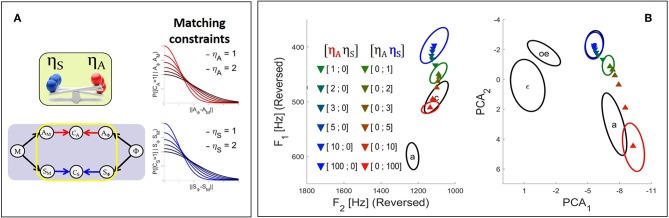
**(A)** Illustration of the effect in the Comparison-based approach of parameters η_*A*_ and η_*S*_ on their corresponding sensory matching constraints. The smaller the value of η, the sharper the constraint function and the stronger the relative contribution of the corresponding sensory pathway to the planning process. **(B)** Results of the fusion planning process after adaptation to the auditory perturbation described in section 2.2.2, for different values of parameters η_*A*_ and η_*S*_.

In the Comparison-based approach we modulate the involvement of each sensory modality at the level of the comparison between sensory-motor predictions and sensory characterizations of phonemes, as illustrated on the left panel of Figure 5. To do so, we have to slightly modify the definition of the operator that performs the comparison, i.e., the sensory matching constraint defined in Equation (4). Until now we have defined the sensory matching constraint in an “all-or-nothing” manner, where terms are either “1” when values of the variable predicted with the sensory-motor map match exactly the sensory-phonological variables, or “0” when they differ, regardless of the magnitude of the difference (see Equation 4). This definition is very strict, as it requires an extreme accuracy in the achievement of the speech motor task in the sensory domain. Intuitively, if we are able to soften this constraint, we may be able to modulate the strengths of the comparisons and hence the involvement of each sensory pathway in the planning process.

We relax the sensory-matching constraint by extending its definition given in Equation (4) as follows (Bessière et al., 2013):

(11) $$\begin{array}{r} P\left(\left[C_{X}=1\right]\;|\;\left[X_{M}=x_{1}\right]\;\left[X_{\Phi }=x_{2}\right]\right)=e^{-d_{X}\left(x_{1},x_{2}\right)}. \end{array}$$

Here *d*_*X*_(*x*_1_, *x*_2_) is a distance measure between sensory values *x*_1_ and *x*_2_. Since *e*^−*x*^ is a decreasing continuous function of *x*, the function defined in Equation (11) gives high probability of matching for *x*_1_ and *x*_2_ values that are close (small distance *d*_*X*_(*x*_1_, *x*_2_)) and low probability of matching for values that are far from each other. Note that the definition given in Equation (4) can be considered to be a degenerate case of this new expression of the sensory-matching constraint, in which the distance measure would be zero when *x*_1_ = *x*_2_ and infinite otherwise. For computational reasons, we choose a distance measure that is quadratic, i.e., $d_{X}\left(x_{1},x_{2}\right)={\left(x_{1}-x_{2}\right)}^{2}$. This choice enables to obtain a closed analytic form for the derivation of the motor planning question.

With this new expression of the matching constraint, we implement sensory preference in the model by introducing two additional parameters, respectively η_*A*_ and η_*S*_, for the auditory and the somatosensory pathway. These parameters modulate the sensitivity of the distance measures *d*_*A*_(*a*_1_, *a*_2_) and *d*_*S*_(*s*_1_, *s*_2_) associated with the sensory pathways:

(12) $$\begin{array}{r} d_{X}\left(x_{1},x_{2};\eta_{X}\right)=\frac{{\left(x_{1}-x_{2}\right)}^{2}}{2\eta_{X}^{2}}. \end{array}$$

With this choice of parametric quadratic measure, Equation (11) becomes:

(13) $$\begin{array}{r} P\left(\left[C_{X}=1\right]\;|\;\left[X_{M}=x_{1}\right]\;\left[X_{\Phi }=x_{2}\right]\right)=e^{-\frac{{\left(x_{1}-x_{2}\right)}^{2}}{2\eta_{X}^{2}}} \end{array}$$

Figure 5A illustrates the form of the matching constraint defined by Equations (13) in the Comparison-based approach for different values of parameter η_*X*_: small values of η_*X*_ lead to sharper matching constraints; large values lead to flatter constraints. Note in particular that for η_*X*_ → 0 the rigid constraint formulated in Equation (4) is recovered, while for η_*X*_ → +∞ the constraint function becomes constant, independent of the sensory values, which in fact corresponds to an absence of constraint.

## 3. Results

### 3.1. Simulating Sensory Preference

#### 3.1.1. Simulation of the Target-Based Approach

We now illustrate results of simulations using the Target-based approach to model sensory preference in the context of the adaptation to the auditory perturbation described above in section 2.2.2. The colored triangles in Figure 4 present the mean results computed for different values of parameters κ_*A*_ and κ_*S*_ based on 2.10^4^ samples in the motor control space. For reference, colored ellipses present the results obtained with the three planning processes of the previous Section [i.e., purely auditory (red color), purely somatosensory (blue color), or “fusion” planning (intermediate color)].

It can be seen that, as expected, progressively increasing parameter κ_*A*_ leads to results that progressively drift toward the outcome of the pure somatosensory planning process. Similar results are obtained toward the outcome of the pure auditory planning when progressively increasing κ_*S*_. Hence, parameters κ_*A*_ and κ_*S*_ effectively modulate the strength of each sensory pathway. This confirms the possibility of implementing sensory preference in our model in a way similar to previous approaches: modulating the relative precision of sensory target regions effectively modulates the contribution of the corresponding sensory pathway.

#### 3.1.2. Simulation of the Comparison-Based Approach

We now illustrate the Comparison-based approach to model sensory preference, and study the effect of parameters η_*A*_ and η_*S*_ in the model in the context of the adaptation to the auditory perturbation described above in section 2.2.2. The colored triangles in Figure 5 present the mean results computed for different values of parameters η_*A*_ and η_*S*_ based on 2.10^4^ samples in the motor control space. As in Figure 4, colored ellipses present the results obtained with the three initial planning processes, for reference.

It can be seen that progressively increasing parameter η_*A*_ of the auditory matching constraint leads to results that progressively drift toward the outcome of the somatosensory planning process. Similarly increasing parameter η_*S*_ of the somatosensory matching constraint results in a drift toward the outcome of the auditory planning process. Hence, parameters η_*A*_ and η_*S*_ successfully enable to modulate the strength of the constraint imposed by the corresponding sensory pathways.

### 3.2. Equivalence of the Approaches

We have formulated two alternative approaches to implement sensory preference in Bayesian GEPPETO. Although these approaches account for clearly different ways to process sensory variables, simulations with the model have shown that they lead to qualitatively similar results (right panels of Figures 4, 5). Increasing parameter κ_*A*_ or parameter η_*A*_ decreases in a comparable manner the involvement of the auditory modality in the model, and, thus, the magnitude of the changes induced by the compensation for the auditory perturbation. Thus, at the limit, for very large values of κ_*A*_ or η_*A*_, the magnitude of the compensation for the auditory perturbation tends toward zero, which perfectly matches the results of the pure somatosensory planning process. Conversely, increasing parameter κ_*S*_ or parameter η_*S*_ decreases the involvement of the somatosensory modality and induces an increase of the magnitude of the compensation for the auditory perturbation. At the limit, for very large values of κ_*S*_ or η_*S*_, the magnitude of the compensation tends toward the magnitude obtained with the pure auditory planning process.

However, a closer comparison of the results presented in the right panels of Figures 4, 5 reveals differences in the ways the compensation for the auditory perturbation varies when parameters κ_*X*_ or η_*X*_ vary. In the Target-based approach, the sequence of compensatory results follows a slightly more simple and straight path than in the Comparison-based approach.

Despite these slight differences, the qualitative similarity of the results obtained with both approaches can be formally explained. Indeed, let us consider the outcome of the fusion planning *P*([*M* = *m*] | Φ [*C*_*A*_ = 1] [*C*_*S*_ = 1]) using the generalized sensory matching constraints given by Equation (11) in the Comparison-based approach. It yields:

(14) $$\begin{array}{l} P\left(\left[M=m\right]\;|\Phi \left[C_{A}=1\right]\;\left[C_{S}=1\right]\right)\\ \propto \underset{a_{\Phi }}{\sum }P\left(\left[A_{\Phi }=a_{\Phi }\right]|\Phi \right)P\left(\left[C_{A}=1\right]\;|\;\left[A_{\Phi }=a_{\Phi }\right]\;\left[A_{M}=\rho_{a}\left(m\right)\right]\right)\\ \begin{array}{c} \;\underset{s_{\Phi }}{\sum }P\left(\left[S_{\Phi }=s_{\Phi }\right]|\Phi \right)P\left(\left[C_{S}=1\right]\;|\;\left[S_{\Phi }=s_{\Phi }\right]\;\left[S_{M}=\rho_{s}\left(m\right)\right]\right), \end{array} \end{array}$$

where we have omitted intermediate steps for the sake of brevity. Now, using the definition of sensory targets given in Equation (2) and the quadratic distance in the matching constraints as given in Equation (13), we note that all terms on the right hand side of Equation (14) are Gaussian. Hence, we can rewrite Equation (14) as:

(15) $$\begin{array}{l} P\left(\left[M=m\right]\;|\;\Phi \;\left[C_{A}=1\right]\;\left[C_{S}=1\right]\right)\\ \propto \underset{a_{\Phi }}{\sum }N\left(a_{\Phi };\mu_{A}^{\Phi },\;\Gamma_{A}^{\Phi }\right)N\left(a_{\Phi };\rho_{a}\left(m\right),\eta_{A}^{2}I_{A}\right)\\ \begin{array}{c} \;\underset{s_{\Phi }}{\sum }N\left(s_{\Phi };\mu_{S}^{\Phi },\;\Gamma_{S}^{\Phi }\right)N\left(s_{\Phi };\rho_{s}\left(m\right),\eta_{S}^{2}I_{S}\right), \end{array} \end{array}$$

where we have denoted by *I*_*A*_ and *I*_*S*_ the identity matrices in the auditory and somatosensory space, respectively. With the introduction of variable *y* = ρ_*x*_(*m*)−*x*_Φ_, each of the sums in Equation (15) are in fact the convolution of two Gaussian distributions, one with mean $\mu_{X}^{\Phi }$ and covariance $\Gamma_{X}^{\Phi }$, the other of mean 0 and covariance $\eta_{X}^{2}I_{X}$. The convolution of two Gaussian distributions with mean vectors μ_1_, μ_2_ and covariances Σ_1_, Σ_2_ is known to result in another Gaussian distribution with mean vector μ_1_+μ_2_ and covariance Σ_1_+Σ_2_. Hence, the planning process becomes:

(16) $$\begin{array}{l} P\left(\left[M=m\right]\;|\;\Phi \;\left[C_{A}=1\right]\;\left[C_{S}=1\right]\right)\\ \begin{array}{c} \propto N\left(\rho_{s}\left(m\right)\;;\;\mu_{S}^{\Phi },\;\Gamma_{S}^{\Phi }+\eta_{S}^{2}I_{S}\right)N\left(\rho_{a}\left(m\right)\;;\mu_{A}^{\Phi },\;\Gamma_{A}^{\Phi }+\eta_{A}^{2}I_{A}\right). \end{array} \end{array}$$

Let us compare Equation (16) and Equation (10): they are almost identical, except for the form of the covariance matrices in auditory and somatosensory spaces. The planning process in the Target-based approach (Equation 10) involves Gaussian distributions with covariance matrices that are modulated multiplicatively by the parameters κ_*A*_ and κ_*S*_, whereas the planning process in the Comparison-based approach (Equation (16)) involves Gaussian distributions with covariance matrices that are modulated additively by parameters η_*A*_ and η_*S*_. Hence, the effect of parameters η_*X*_ and κ_*X*_ are qualitatively similar, as we have illustrated experimentally: they both induce an increase in the covariance of the sensory characterization of phonemes. However, quantitatively, we have shown that parameters κ_*X*_ increase them multiplicatively, whereas parameters η_*X*_ increase them additively.

We note that if the auditory and somatosensory spaces would be one-dimensional, both approaches would be exactly equivalent, since any additive increase Γ+η can be written as a multiplicative increase κΓ, with $\kappa =1+\frac{\eta }{\Gamma }$. This is not true anymore in higher dimensions though, since the Target-based approach scales all coefficients of the covariance matrices, whereas the Comparison-based approach only modifies their diagonal terms. More specifically, the Target-based approach increases the size of the target regions while preserving their orientation, whereas the Comparison-based approach stretches the regions along the coordinate axes, inducing a progressive alignment of the main axes of the target regions with the coordinate axes (off-diagonal terms in the covariance matrices become negligible compared to the increased diagonal terms, and the resulting ellipsoid regions progressively lose their orientations). We assume that the slight differences observed above in the consequences on compensation of progressive variations of the κ_*X*_ and η_*X*_ parameters find their origins in these changes in target orientations.

**Figure 6:**
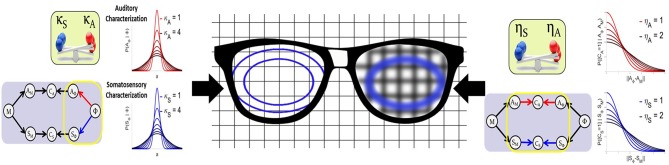
Illustrative interpretation of the equivalence between the two implementations of sensory preference. In the Target-based approach (left part of the figure) the size of the auditory or somatosensory target regions are directly modified with parameters κ_*A*_ and κ_*S*_. In the Comparison-based approach (right part of the figure) parameters η_*S*_ and η_*A*_ modulate the sensitivity of the corresponding sensory matching constraint, as if target regions were “blurred,” making them appear larger.

Figure 6 gives an intuitive interpretation of the equivalence of these two approaches. On the one hand, the Target-based approach directly modulates the size of the target regions, while keeping their orientations, as illustrated on the left lens of the glasses in Figure 6. On the other hand, the Comparison-based approach does not change the targets, but modifies the precision of the comparison of the target with the sensory-motor predictions. This is as if the target were seen through a blurring lens, that would “spread” the borders of the target, making it appear bigger. This “blurring effect” is induced by the convolution of the target with a Gaussian term that acts as noise (Equation 15). The larger the value of parameter η_*X*_, the larger the power of the noise, and the stronger the “blurring” of the target.

## 4. Discussion

The main contribution of our work is to present two different approaches implementing sensory preference in a speech production model that integrates both the auditory and the somatosensory modality. This is done in the context of our Bayesian GEPPETO model for speech motor planning and speech motor control (Perrier et al., 2005; Patri et al., 2016; Patri, 2018), which specifies both auditory and somatosensory constraints to infer motor commands for the production of a given phoneme. We have implemented sensory preference in this model by modulating the relative involvement of sensory modalities with two different approaches: (1) the Target-based approach, which modulates the precision of auditory and somatosensory target regions; (2) the Comparison-based approach, which modulates the sensory-matching constraints between predictions from internal models and sensory target regions. At the core of the evaluation of the two approaches, we have considered the phenomenon of incomplete compensation for sensory perturbations in speech production and its inter-subject variability, which has been evidenced by several experimental studies. Although conceptually different, we have shown in our model that these two approaches are able to account for incomplete compensation variability under the same amount of change in the internal model resulting from adaptation. Furthermore, we have demonstrated the mathematical equivalence of the two approaches in some specific cases, which explains the qualitative similarity of results obtained under both approaches.

In this context, the main outstanding question is whether the two modeling variants are distinguishable. We consider two aspects of this issue: mathematical formulation and experimental evaluation.

Let us compare the mathematical formulations of the two approaches. The Comparison-based approach is less compact and contains more degrees-of-freedom than the Target-based approach. We have also demonstrated that, under certain assumptions, both models behave similarly. On parsimony grounds, then, the Target-based approach certainly wins over the Comparison-based approach. On the other hand the additional degrees of freedom enable the Comparison-based approach to be more flexible.

For further experimental evaluation we consider two possible directions. First, our simulation results illustrate that the particular pattern of partial compensation obtained under both approaches slightly differ. Whether and how these differences could be assessed experimentally is an open question. The main difficulty arises from the fact that the observed differences in partial compensation do not only depend on differences in compensation mechanisms induced by each approach, but also on speaker specific relations between motor commands and sensory variables. Taking into account these speaker specific characteristics would be the main challenge in this experimental evaluation.

The second direction for experimental evaluation, would be related to the different flexibility associated with each approach. Whereas the Target-based approach would predict fixed compensation strategies, ascribing any remaining variability to causes unrelated to sensory preferences or measurement errors, the Comparison-based approach would potentially relate sensory preference with some aspects of the structure of the observed variability. Furthermore, experimentally induced effects (e.g., asking subjects, for a given trial block, to focus especially on somatosensation; introducing a dual-task condition to induce attentional load, etc.) could help discriminating between the predictions of the two models.

Overall, the results of our study provide a new contribution to the understanding of the sensory preference phenomenon. They highlight that two factors could influence sensory preference, that mostly differ by their temporal stability. On the one hand, the Target-based approach represents sensory preference as the precision of target regions. This suggests that sensory preference is learned through language interaction and is stable over time, as the target regions would be used during everyday speech planning. On the other hand, the Comparison-based approach represents sensory preference “elsewhere” in the model, so that it can mathematically be manipulated independently of sensory target regions. Indeed, in this second approach, we have explicitly considered two independent components: (1) the sensory characterization of phonemes, which are mathematically characterized as constraints via the specification of sensory target regions; (2) matching-constraints, which modulate the precision with which sensory predictions from the internal models are compared with phoneme related sensory target regions. This allows a more general and flexible model, as compared to the Target-based approach. This flexibility suggests ways in which sensory preference would be modulated by cognitive control or attentional processes. Such an attentional model would explicitly modulate on the fly sensory preference depending on the context. This modulation could arise, for example, from changes in the access to one of the sensory modality due to disorders, aging, or noise, or from the absence of congruence between the two sensory pathways. A proposal for such an attentional model, as an extension of the Comparison-based model presented here, is outlined in Supplementary Material.

Finally, we turn to possible theoretical extensions and applications of our model. So far, the Comparison-based approach of sensory preference we have described here is constrained by the specific hypotheses of the Bayesian-GEPPETO model in which it is included. For instance, it only concerns sensory preference between somatosensory and acoustic descriptions of targets during serial order planning of sequences of vocalic speech sounds. Of course, the application scope could be extended, e.g., toward sensory preference during movement execution and movement correction, with a finer temporal resolution than we have considered so far. This would for instance allow to study time-varying sensory preference, or sensory preference that depends on speech sounds. Indeed, it is an open question whether consonant and vocalic sounds would differ on the sensory pathway they more precisely rely on. We could also consider using our Comparison-based architecture for describing how low-level sensory acuity would affect the learning of the target representations, and how different sensory preference during this learning would result in different sizes and separations of targets in each sensory pathway. Finally, such a learning mechanism with individual-specific sensory preference could contribute to the emergence of learned idiosyncrasies.

Furthermore, to put our approach in a wider theoretical context, we observe that the Comparison-based approach has a structure that could be cast into the general predictive coding framework, as popularized recently by the free-energy principle proposal (Friston and Kiebel, 2009; Feldman and Friston, 2010; Friston, 2010). Indeed, even though our model does not represent time or time-delays specifically, it nevertheless features the idea that “predictions” from internal models would be compared with sensory targets. We note that this is not exactly the same situation as for a comparison between forward predictions and sensory feedback, as would be used for instance in models of trajectory monitoring; nevertheless, the architecture is similar. In the Comparison-based approach, we have proposed a mathematically specific expression of the “comparison” operator, using probabilistic coherence variables and match measures. Whether this would be a plausible, or at least useful mathematical implementation of probabilistic comparison in predictive coding or free-energy architectures is an open question.

## Data Availability Statement

The datasets generated for this study are available on request to the corresponding author.

## Author Contributions

J-FP, JD, and PP contributed conception and design of the study, and revised the manuscript. J-FP implemented the model and performed simulations, and wrote the first draft of the manuscript. All authors contributed to manuscript revision, read, and approved the submitted version.

### Conflict of Interest

The authors declare that the research was conducted in the absence of any commercial or financial relationships that could be construed as a potential conflict of interest.
