# Supplementary material for: Modeling Sensory Preference in Speech Motor Planning: A Bayesian Modeling Framework
Source: Front Psychol. 2019 Oct 25;10:2339. doi: 10.3389/fpsyg.2019.02339 (PMC6824204; doi:10.3389/fpsyg.2019.02339)
Supplement: Supplementary file 1 [file Data_Sheet_1.PDF]

# Joint control of sensory-matching constraints: towards a modality-weighing attentional model

In this Supplementary Material, we outline a proposal definition of an attentional model, allowing to modulate, on the fly, the relative preference on each sensory modality. To develop this definition, we exploit one of the properties of Bayesian algorithmic modeling (Diard, 2015): using the probabilistic mathematical framework facilitates extending models in a modular fashion. For instance, the Bayesian GEPPETO model we have presented so far is actually a portion of a more extended model, which also includes and represents effort constraints during motor planning (Patri et al., 2016; Patri, 2018).

We remark that Eq (13) of the main text involves parameters η_A and η_S. We rewrite it, with these parameters appearing as explicit probabilistic variables:

$$P([C_S = 1] \mid [S_M = s_1] \; [S_\Phi = s_2] \; \eta_S) = e^{-\frac{(s_1 - s_2)^2}{2\eta_S^2}} \quad \text{(S1)}$$

$$P([C_A = 1] \mid [A_M = a_1] \; [A_\Phi = a_2] \; \eta_A) = e^{-\frac{(a_1 - a_2)^2}{2\eta_A^2}} . \quad \text{(S2)}$$

To be complete, the joint probability distribution of the model is rewritten to include probability distributions about variables η_S and η_A. To define an attentional model, that involves some balance constraint between η_S and η_A, we further introduce another probabilistic variable, which we note ζ. Eq (1) of the main text becomes (see Figure S1):

$$\begin{aligned} P(M \; \Phi \; A_M \; A_\Phi \; C_A \; S_M \; S_\Phi \; C_S \; \eta_A \; \eta_S \; \zeta) = \; & P(M)P(\Phi) \\ & P(A_M \mid M)P(A_\Phi \mid \Phi)P(C_A \mid A_M \; A_\Phi \; \eta_A) \\ & P(S_M \mid M)P(S_\Phi \mid \Phi)P(C_S \mid S_M \; S_\Phi \; \eta_S) \\ & P(\eta_A \mid \zeta)P(\eta_S \mid \zeta)P(\zeta) . \end{aligned} \quad \text{(S3)}$$

To complete the mathematical definition of the model, probability distributions $P(\eta_A \mid \zeta)$, $P(\eta_S \mid \zeta)$ and $P(\zeta)$ have to be defined. For instance, an attentional model would amount to establishing a constraint, such that processing has to be spread over both sensory modalities, maybe even forbidding both constraints to be “fully taken into account” simultaneously. This would be piloted by the value of the ζ variable, which would then represent a distribution of attentional resource across sensory modalities, providing the means to flexibly modulate sensory preference in the model, in a volatile, possibly trial-to-trial varying manner (if it was itself piloted by other, top-down constraints, variable ζ could vary over time).

Any number of mathematical formulations could be considered, that would comply with the above description, leading to different implementations of the attentional model. For instance, a specific implementation could consist of constraining the sum of the values of η_A and η_S to be constant, so that increasing one parameter decreases the other, with variable ζ representing their balance. Assume for instance the sum to be 10, an equilibrium would be to have $\eta_S = \eta_A = 5$, and increasing the force of the auditory constraint ($\eta_A = 2$) would decrease the force of the somatosensory constraint ($\eta_S = 8$). This example yields an “additive” and linear attentional scale, with a particular equilibrium point and with a

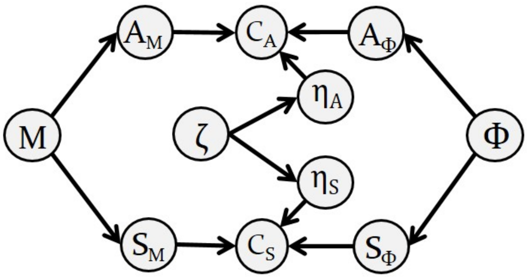

Figure S1. Diagram representing the architecture of the model with sensory preference modulated by an attentional process in the context of the Comparison-based approach to model sensory preference. The diagram is a graphical representation of the decomposition of the joint probability distribution given in Eq (S3).

particular balance (to have one constraint be "fully taken into account", i.e., around 0, the other has to be close to 10), etc.

Simulating such a model is straightforward, and results are as expected from previous simulations. Indeed, when η_A and η_S are jointly modified in this manner, simulations lead to adaptation results intermediary between the $[\eta_A = 10, \eta_S = 0]$ and the $[\eta_A = 0, \eta_S = 10]$ simulations shown Figure 5 of the main text.

REFERENCES

Diard, J. (2015). *Bayesian Algorithmic Modeling in Cognitive Science*. Habilitation à diriger des recherches (HDR), Université Grenoble Alpes

Patri, J.-F. (2018). *Bayesian modeling of speech motor planning: variability, multisensory goals and perceptuo-motor interactions*. Unpublished Ph.D. thesis, Université Grenoble Alpes

Patri, J.-F., Perrier, P., and Diard, J. (2016). Bayesian modeling in speech motor control: a principled structure for the integration of various constraints. In *Interspeech 2016* (San Francisco), 3588–3592
